# Effects of plant growth regulator treatments on quality-related traits and the establishment of cultivar-specific cultivation conditions in small- and medium-sized lily bulbs

**DOI:** 10.3389/fpls.2026.1916678

**Published:** 2026-08-19

**Authors:** Kyong-Eun Jeon, Hye-Gyeong Hyeon, Seong Heo

**Affiliations:** 1 Department of Horticulture, Kongju National University, Yesan, Republic of Korea; 2 Chungcheongnam-Do Agricultural Research and Extension Services, Yesan, Republic of Korea

**Keywords:** 6-benzylaminopurine, cultivar-specific cultivation, cytokinin, flower bud number, lily bulbs, plant growth regulators, response surface methodology

## Abstract

High-quality lilies with multiple flower buds are in demand for export, but small- and medium-sized bulbs often produce fewer buds, prompting the need for improved cultivation strategies. In this study, the effects of pre-planting plant growth regulator treatments on cut flower quality (flower bud number and length, plant height, and bulb circumference) in small- and medium-sized lily bulbs (14–16 cm) were investigated. Six lily cultivars (‘Aubisque,’ ‘Difference,’ ‘Ducati,’ ‘Spring City,’ ‘Sweet Zanica,’ and ‘Woori Tower’), representing three lineages, were treated with 6-benzylaminopurine (BA) or gibberellin (GA) + BA at different concentrations and immersion times. Immersion in BA for 30 min significantly increased flower bud number overall (*p* = 0.0003), with 0.20 mM identified as the most effective concentration, but the response was strongly cultivar dependent: a significant increase occurred in only two of the six cultivars (‘Spring City’ and ‘Sweet Zanica’), the remaining four were unresponsive, and GA+BA was ineffective in all cultivars. Machine learning models were constructed to predict flower bud number (R² = 0.558) and bulb circumference (R² = 0.915) from phenotypic traits. Correlation and principal component analysis identified shoot fresh weight and number of stem leaves as the grower-observable traits most closely associated with flower bud number, and response surface methodology was used to convert them into cultivar-specific growth targets. Pre-planting BA immersion therefore offers an economic and practical strategy for producing export-grade cut flowers from small- and medium-sized bulbs, but only in cytokinin-responsive cultivars, which must be identified before commercial adoption.

## 1 Introduction

Lilies (*Lilium* spp.), a well-known ornamental bulbous plant with diverse colors and fragrances, are a key component of the global ornamental plant market as both cut flowers and potted plants (Grassotti and Gimelli, 2011). In South Korea, lilies are the third most produced cut flower crop, following roses and chrysanthemums, accounting for 76 ha (6.3%) of the 1,208 ha of the cut flower cultivation area and USD 1.063 million, equivalent to 11.4% of the total export revenue of USD 9.313 million (MAFRA, 2024). Several commercially important lily species are cultivated for cut flower production. Oriental hybrids (O), characterized by their striking flowers and pleasant fragrance, account for 50% of the commercial market, followed by *Lilium longiflorum* (L), with pure white, trumpet-shaped flowers, and *L. longiflorum* × Asiatic hybrids (LA hybrids), which have diverse colors and upward-facing flowers (Kim and Kim, 2015). Oriental hybrids, while highly preferred by consumers, can be difficult to cultivate because of their susceptibility to viruses. To address this limitation, Oriental hybrids have been crossed with trumpet hybrids to produce Oriental × trumpet hybrids (OT hybrids), which exhibit robust growth and strong pest tolerance (Lee et al., 2019). Recently, OT hybrids have been cultivated in South Korea to replace virus-vulnerable Oriental hybrids.

Compared with major exporters of lilies, such as the Netherlands and China, South Korea’s geographical proximity to Japan gives it a competitive edge in terms of cut flower freshness and reduced logistics costs. This advantage is further reinforced by the decline in domestic lily production in Japan owing to population aging. In summer, when the environment in Japan is hot and humid, high-quality lilies produced in the cool, high-altitude regions of Gangwon-do in South Korea are imported, accounting for over 90% of the Japanese lily import market. The predictable demand for cut lilies in Japan enables stable exports from lily-producing farms in South Korea (Kang et al., 2009).

The standards for high-quality lilies preferred by Japanese consumers include vivid flower color, a clean external appearance with no pest damage, a plant height of ≥90 cm, excellent freshness, and long vase life. Flower bud number is a key quality index for determining cut flower quality (Li et al., 2023), with ≥5 buds considered premium quality (Ko et al., 2013). As a higher number of buds per stem enhances perceived floral quality, producing lilies with at least five flower buds is a key objective for export to Japan.

The number of flower buds in lilies has been studied since the 1970s, with bulb size identified as a key determining factor (Suh et al., 2013). In a study on OT hybrids by Lee et al. (2020), a bulb circumference of 18–20 cm resulted in an average flower bud number of 4.2, and a bulb circumference of ≥20 cm was associated with an average flower bud number of 6.4. Similarly, in LA hybrid lilies, an average flower bud number of >4 has been reported for lilies with a bulb circumference of at least 18–20 cm (Lee et al., 2016, 2018). Lily bulbs act as storage organs that accumulate nutrients for initial shoot growth and subsequent flower production, and bulbs with a large circumference have been linked to increased plant height, flower bud number, offset count, and flower size (Lazare et al., 2019). Generally, to produce high-quality lilies with multiple flowers, large bulbs with a circumference of at least 18–20 cm are required. However, these large bulbs are more expensive and increase the management burden for lily-growing farms. Increasing demand from lily producers for techniques to produce premium cut flowers from small- and medium-sized bulbs (14–16 cm) has prompted several countries, including the Netherlands, to develop methods to improve quality-related traits such as flower bud number. Therefore, developing cultivation techniques to produce export-quality lilies using cost-effective, small- and medium-sized bulbs has become an urgent priority.

Importantly, previous studies have predominantly focused on post-planting application of gibberellin (GA) and the cytokinin 6-benzylaminopurine (BA) by foliar spray or drench, while systematic investigation of pre-planting bulb immersion remains limited. Recent synthesis of the literature on meristem-derived organ initiation in bulbous species confirms this asymmetry: the available evidence on hormonal regulation derives largely from tissue culture systems and from foliar application, and the effects of pre-planting treatment of the propagule itself have not been systematically compared across genotypes (Shu et al., 2024). This gap is consequential, because pre-planting immersion differs from foliar application in the developmental window it targets, acting on the bulb before shoot emergence and therefore before floral primordium initiation, and because the response of a genotype to one delivery route need not predict its response to the other.

Plant growth regulators (PGRs) play a role in improving quality and stress tolerance in crops (Zhang et al., 2024). For ornamental plants, PGRs are used commercially in various ways to improve quality, including promoting longer stems, increasing leaf area, and extending cut flower vase life (Al-Ajlouni et al., 2023). PGR treatment increases flower bud number in ornamental bulbous plants (Sajid et al., 2009; Emami et al., 2011; Sajjad et al., 2014; Al-Ajlouni et al., 2023) through positive effects on bulb development, nutrient accumulation, and flower bud differentiation and development (Salachna et al., 2020). BA is involved in various physiological processes, including the promotion of cell differentiation (Wu et al., 2021), induction of lateral bud development, and delay of senescence (Al-Ajlouni et al., 2023). Furthermore, BA treatment effectively increased lateral bud formation and offset growth in bulbous ornamental plants, including lilies (Takayama and Misawa, 1982). GAs play a central role in increasing stem, leaf, and root length and are reportedly effective at improving flower color and increasing flower bud number (Zhang et al., 2022). In lily, GA_4 + 7_ applied at the visible bud stage accelerated bud elongation and advanced flowering (Hatamzadeh et al., 2010), and gibberellic acid applied to the growing crop has been reported to improve flower quality in lily and gerbera (Othman et al., 2021); both interventions act after floral initiation is complete and therefore on the expansion rather than the initiation of floral organs. This distinction is central to the present study, in which the propagule is treated before shoot emergence. Recently, a growing number of studies have applied a mixture of cytokinins and GAs as PGRs; however, most have focused on GA+BA treatment after bulb planting, with relatively limited research on pre-planting PGR treatments. In addition, the response of plants to PGRs can markedly differ depending on the treatment concentration, time, method, and genotypes of cultivars (Han, 2001). In lilies, numerous lines and cultivars have been developed, and differences in physiological properties among cultivars may result in variable responses to the same PGR. Therefore, to evaluate the effects of specific PGRs and apply them as practical cultivation technologies, systematic research must be conducted across different cultivars.

In this study, we aimed to use small- and medium-sized lily bulbs to produce high-quality lilies suitable for export. To this end, we evaluated various factors, including lily cultivar, PGR type and concentration, and immersion time, to determine whether pre-planting PGR treatment can raise cut flower quality from small- and medium-sized bulbs to the absolute thresholds specified by the Japanese export grade, in particular the premium standard of at least five flower buds (Section 3.1–3.4). Large bulbs were not included as a treatment; the comparison made here is therefore against the export specification itself rather than against a concurrently grown large-bulb crop. In addition, several growth-related characteristics were investigated, including vegetative traits (plant height, stem thickness, leaf length, leaf width, number of stem leaves, fresh weight, and dry weight) and flowering traits (flower bud number, bud length, peduncle length, and flowering width). Based on these phenotypic characteristics, machine learning models were developed to predict quality-related traits (flower bud number, flower bud length, plant height, and bulb circumference; Section 3.5). Finally, to identify optimal cultivation conditions for each cultivar and maximize flower bud number in small- and medium-sized lily bulbs, response surface methodology (RSM) was applied using fresh weight and the number of stem leaves as key predictors, which were strongly correlated with the bud number (Section 3.6).

We emphasize at the outset that the modeling component of this study was not designed as a stand-alone forecasting tool. It was designed for two specific purposes: first, to rank the full set of measured vegetative and floral traits according to their association with export quality traits, thereby identifying the small number of grower-observable traits that carry most of the information about flower bud number; and second, to convert those traits into cultivar-specific growth targets that can be used for in-season crop assessment and for allocating lots between export and domestic channels. The value of this framework lies in the reduction of a 24-trait phenotypic dataset to a small set of interpretable, cultivar-resolved management targets, rather than in high-precision prediction of individual bud counts.

The findings of this study are expected to alleviate the economic burden associated with using expensive large bulbs and provide practical cultivation techniques that growers can directly implement in the field, thereby contributing to enhanced export competitiveness and reduced production costs for lily cultivation. Furthermore, the approach used in this study to derive cultivar-specific optimal conditions can be applied to the development of cultivation techniques for other ornamental bulbous crops.

## 2 Materials and methods

### 2.1 Plant materials and cultivation environment

In this study, six lily cultivars from different lines were used. The Oriental (O) cultivars ‘Aubisque’ (AB) and ‘Spring City’ (SC), LA hybrids (LA) ‘Ducati’ (DU) and ‘Sweet Zanica’ (SZ), and *L. longiflorum* (L) cultivars ‘Difference’ (DI) and ‘Woori Tower’ (WT) are important export cultivars in South Korea and are highly preferred by consumers in the Japanese ornamental plant market. The lineage assignment of each cultivar is summarized in **Table 1**.

**Table 1:** Lineage classification and abbreviations of the six lily cultivars used in this study.

Lineage	Cultivar	Abbreviation
Oriental hybrid (O)	‘Aubisque’	AB
Oriental hybrid (O)	‘Spring City’	SC
*L. longiflorum* × Asiatic hybrid (LA)	‘Ducati’	DU
*L. longiflorum* × Asiatic hybrid (LA)	‘Sweet Zanica’	SZ
*Lilium longiflorum* (L)	‘Difference’	DI
*Lilium longiflorum* (L)	‘Woori Tower’	WT

The lily bulbs were produced by a bulb production company in the Netherlands (Onings, Netherlands; C. Steenvoorden B.V., the Netherlands). Small- and medium-sized bulbs (14–16 cm) were selected and purchased from a South Korean bulb company (Woori Seed/Woori Flower Seed & Seedling Co., Ltd., Gwacheon-si, South Korea). After harvesting at the Dutch production farm, the bulbs were refrigerated (4 °C) during transport to South Korea. The bulbs were visually inspected immediately after arrival, and only undamaged, healthy bulbs were selected.

For the experiment, lilies were cultivated in a plastic greenhouse within the experimental farm at the Chungcheongnam-do Agricultural Research and Extension Services Flower Research Institute. On March 25, 2024, the bulbs were planted in square plastic pots (63.5 × 24 × 17 cm, L × W × H) filled with 100% coir. In terms of plant density, 1,800 bulbs were planted at 10 × 15 cm intervals, with 5 bulbs allocated per treatment for each cultivar. During the cultivation period, the greenhouse environment was controlled to an average daytime temperature of 25–28 °C and an average nighttime temperature of 15–18 °C to provide optimal growth conditions for the lilies. These conditions were consistent with the recommended temperatures for lily cultivation (25 °C during the day and 15 °C at night) (Rural Development Administration, 2020). Humidity was controlled at 60 ± 10%, and ventilation fans and side windows were used to regulate temperature and humidity.

Irrigation was conducted using a drip line. To promote root attachment after planting, irrigation was conducted once every 7 d for the first 2 weeks. From the mid-growth stage, irrigation was conducted once every 3 d based on soil moisture conditions. A compound fertilizer (N19-P19-K19, Poly-Feed; Haifa Chemicals, Haifa, Israel) was diluted 1,000-fold to a concentration of less than EC 0.5 dS·m^-1^ and delivered through the drip line once per week. For pest and disease control, a rotational pesticide program was implemented to prevent resistance development. Soil insecticides were applied as soil drenches at 2-week intervals, rotating among Conido (imidacloprid 8% SC; Bayer CropScience Korea, Seoul, South Korea), Baetada (etofenprox 0.5% + terbufos 3%; Sungbo Chemicals, Seoul, South Korea), Mocap (ethoprophos 5%; Nonghyup Chemical, Seoul, South Korea), The One (benfuracarb 30% WG; Dongbang Agro, South Korea), and Movento (spirotetramat 225 g/L SC; Bayer CropScience Korea). Fungicides Captan (captan 50% WP; Farm Hannong, Seoul, South Korea) and Benomyl (benomyl 50% WP; Farm Hannong, Seoul, Korea) were applied as foliar sprays at 2-week intervals. When pests were detected, foliar insecticides, including Delegate (spinetoram 5% WG; Farm Hannong), Dolgyeokdae (dichlorvos 20% + lambda-cyhalothrin 0.8% DC; Farm Hannong), or Fanfare-S (pyrifluquinazon 6.5% SC; Kyung Nong, Seoul, South Korea) were additionally applied following visual diagnosis. All pesticides were diluted to the concentrations recommended on the product labels.

### 2.2 PGR treatment

The cytokinin BA (6-BA, purity 98%; Yooill, Seoul, South Korea) and a commercial formulation containing 1.8% GA_4 + 7_ and 1.8% 6-BA (Pomina; Nonghyup Chemical; South Korea; hereafter referred to as GA+BA) were used. BA solutions were prepared at concentrations of 0, 0.10, 0.15, 0.20, and 0.25 mM by dissolving BA in distilled water. The commercial GA+BA formulation was diluted with distilled water to nominal total molar concentrations of 0, 0.07, 0.13, 0.20, and 0.27 mM. The molar concentration of each component delivered by the mixed formulation is therefore given in **Table 2**. Notably, the BA delivered at the highest GA+BA concentration tested was 0.161 mM, which is below 0.20 mM, the lowest BA concentration at which a significant increase in flower bud number was observed when BA was applied alone. All solutions were prepared by completely dissolving the compounds in distilled water, and distilled water alone was used as the control. Concentrations were labeled by indicating the PGR type (BA or GA+BA), the concentration value, and “C” to denote concentration.

**Table 2:** Molar concentrations of the active components delivered by each plant growth regulator treatment.

Treatment	Nominal concentration (mM)	GA_4 + 7_ (mM)	6-BA (mM)
Control	0	0	0
BA	0.10	0	0.10
BA	0.15	0	0.15
BA	0.20	0	0.20
BA	0.25	0	0.25
GA+BA	0.07	0.028	0.042
GA+BA	0.13	0.052	0.078
GA+BA	0.20	0.081	0.119
GA+BA	0.27	0.109	0.161

Concentrations of the mixed formulation refer to the combined molar concentration of GA_4 + 7_ and 6-BA; because the two active ingredients are present in a 1:1 ratio by mass and differ in molar mass, 6-BA accounts for approximately 60% of the molar total. GA, gibberellin; BA, benzylaminopurine.

To select the optimal immersion times for PGRs, bulbs from the six cultivars were completely submerged in 200 L of treatment solution for 2 or 30 min. Each condition was tested in triplicate, and each 200 L of solution contained only one cultivar. Immersion times were labeled by indicating the PGR type (BA or GA+BA), followed by the duration and “I” to denote immersion.

After immersion, the bulbs were air-dried in the shade for approximately 2 h at room temperature (20 ± 2 °C) and then immediately planted. All treatments were performed in triplicate, and different containers were used for each treatment to prevent cross-contamination.

### 2.3 Lily growth and flowering characteristics

#### 2.3.1 Bulb characteristics before planting

The lily bulb characteristics of the six cultivars were studied before planting. The variables studied included bulb height, circumference, weight, and the number and length of roots. Bulb circumference was measured at the widest point using a tape measure, and bulb weight was measured to the nearest 0.1 g using an electronic scale (MW-II-300; MIGUN ST, Siheung, South Korea). The number of roots was determined by counting all roots on each bulb before PGR treatment, and root length was calculated as the mean of the three longest roots.

#### 2.3.2 Shoot system growth and flowering characteristics

Shoot system growth and flowering characteristics were investigated at the flowering time of each cultivar. Flowering characteristics were assessed when the first bud of each cultivar had fully opened. The variables evaluated for growth characteristics included plant height (cm), stem thickness (mm), leaf length (cm), leaf width (cm), number of stem leaves (No.), fresh weight (g), and dry weight (g). Plant height was measured from the crown to the tallest inflorescence using a tape measure, and stem thickness was measured using digital vernier calipers (500-182-30; Mitutoyo, Kanagawa, Japan) by recording the stem diameter at 10 cm above the ground. Leaf length and width were measured at their respective maxima from an arbitrarily selected, fully opened leaf in the middle of the plant body. The number of stem leaves on each specimen was determined by counting all leaves from the crown to the highest inflorescence. Fresh weight was measured as the total weight of the shoot system after cutting flowers from 5 cm above the crown, whereas dry weight was determined by weighing after drying for 72 h at 80 °C in a dryer (SteD-30; DAIHAN, Wonju, South Korea).

The variables investigated for flowering characteristics included the flower bud number (No.), flower bud length (cm), petal length (cm), petal width (cm), sepal length (cm), sepal width (cm), peduncle length (cm), flowering width (cm), days of flowering (d), and days until flowering (d). The flower bud number was measured as the total number of buds that fully developed into flowers. The flower bud length was measured as the length of the first bud that bloomed on each cultivar, which is the standard required in specifications for export to Japan. Flowering width was measured as the maximum diameter of the first flower to completely bloom, and peduncle length was measured as the length from the stem to the calyx. Petal and sepal dimensions and flowering dates were recorded for completeness and were included in the principal component and machine learning analyses, but are not presented separately because they were not components of the export grading criteria.

#### 2.3.3 Bulb characteristics after harvesting

Once all cultivars had finished flowering, the shoot systems were harvested from a height of 5 cm above the ground, and the circumference and weight of the bulbs, collected on October 21, 2024, were measured. The bulbs were carefully excavated from the bed soil, cleaned, and measured after ensuring that they were completely dry. Bulb size and weight after harvesting are important indices for assessing the quality of bulbs as stock bulbs for cultivation in the subsequent year. All measurements were performed in triplicate using five specimens per treatment group, and the timing of measurements was standardized to minimize environmental variations.

### 2.4 Quality grading and frequency analysis

Quality grades for cut lilies for export to Japan are based on criteria established over 3 years by the Center for Research and Development of Lilium, South Korea. The cut flower quality traits used to determine the grade included plant height, flower bud number, flower bud length, and bulb circumference. Grades were assigned separately for each of the four traits. For plant height the thresholds were premium ≥ 90 cm, good 80.1-89.9 cm, and medium ≤ 80cm; for flower bud number, premium ≥ 5, good 4, and medium, <4; for flower bud length, premium ≥ 12cm, good 9.5-11.9 cm, and medium <9.5 cm; and for bulb circumference, premium > 18cm, good 14.0-18.0 cm, and medium < 14cm. A composite grade requiring all four thresholds to be met simultaneously was not computed. For each trait, grade distribution was analyzed to evaluate the characteristics of each cultivar and treatment group, and the proportion of high-quality grades (premium + good) was used as an index of export suitability.

### 2.5 Statistical analysis

The effects of PGR treatments on improving the export quality grades of lilies derived from small- and medium-sized bulbs were analyzed. A factorial analysis of variance was performed with PGR type (BA or GA+BA), cultivar, PGR concentration (0, 0.10, 0.15, 0.20, or 0.25 mM for BA and 0, 0.07, 0.13, 0.20, or 0.27 mM for GA+BA), and immersion time (2 or 30 min) as factors. The main effects of each factor and the interaction effects were analyzed. Shapiro–Wilk and Levene’s tests were used to test the assumptions of normality and homoscedasticity, respectively. Differences in mean values among treatment groups were evaluated using Scheffé’s test for *post-hoc* analysis at a significance level of *p* < 0.05 (Sections 3.1 and 3.2).

To ascertain the quality grade characteristics in accordance with Japanese export specifications, the plants were graded based on the criteria explained in Section 2.4, and the frequency of quality grades was analyzed for each cultivar and treatment group. The proportion of high-quality grades (premium + good) was calculated for each cultivar and treatment group to evaluate suitability for export (Section 3.3).

To investigate the primary growth traits related to flower bud number, principal component analysis (PCA) was performed using R software (R Core Team, 2024) with the FactoMineR (Lê et al., 2008) and factoextra (Kassambara and Mundt, 2020) packages. All investigated traits were analyzed depending on the cultivar, lily type, immersion time, and concentration. The 95% confidence interval in each group was displayed as an ellipse to distinguish differences between treatments (Section 3.4).

To predict cut flower quality characteristics for lilies, a machine learning algorithm was used to construct models to predict plant height, flower bud number, flower bud length, and bulb circumference. Using the h2o package in R (Fryda et al., 2024), regression models were developed to predict each cut flower quality trait. Models were selected by automated machine learning (h2o.automl), which searched over generalized linear models, gradient boosting machines, random forests, extremely randomized trees, deep neural networks and stacked ensembles within a fixed time budget, using 5-fold cross validation and a fixed random seed. To evaluate the prediction performance of the regression-based predictions, the root mean square error, mean absolute error, and coefficient of determination (*R*²) were calculated (Section 3.5).

Two predictor sets were used. The full set comprised all vegetative and floral traits recorded during the experiment together with the treatment factors. The pre-planting set was restricted to information available before crop establishment — cultivar, PGR type, concentration, immersion time, and the bulb attributes measured before planting (bulb circumference, bulb weight, bulb height, number of bulb roots and root length; Section 2.3.1) — and was used to determine the extent to which the decision to apply BA to a given bulb lot can be supported *ex ante*. Model performance was evaluated on cross-validated holdout predictions rather than on training-set predictions. Because the operational decision faced by growers is categorical, namely whether a lot will meet the Japanese premium standard of at least five flower buds, predicted values were additionally evaluated as a binary classifier against that threshold using the pROC package, and the area under the receiver operating characteristic curve, sensitivity and specificity at the cut-off maximizing Youden’s J were computed. Finally, leave-one-cultivar-out cross-validation was performed, in which the model was trained on five cultivars and evaluated on the sixth, to quantify transferability to cultivars not represented in the training data.

To estimate the optimal cultivation conditions for maximizing flower bud number, the correlations between flower bud number and major growth-related traits were analyzed. After identifying significant variables, the rsm package in R was used to implement RSM to determine the optimal cultivation conditions associated with flower bud number for each cultivar (Lenth, 2009; Kim and Heo, 2025). Second-order response surfaces were fitted separately for each cultivar, and predictions were restricted to the range of the observed data to avoid extrapolation.

Graphs were plotted in R 4.5.0 (R Foundation for Statistical Computing, Vienna, Austria) using the following packages: dplyr, ggplot2, emmeans, ggpubr, rstatix, and tidyverse.

## 3 Results

### 3.1 Effects of PGR on quality-related traits

**Figure 1:**
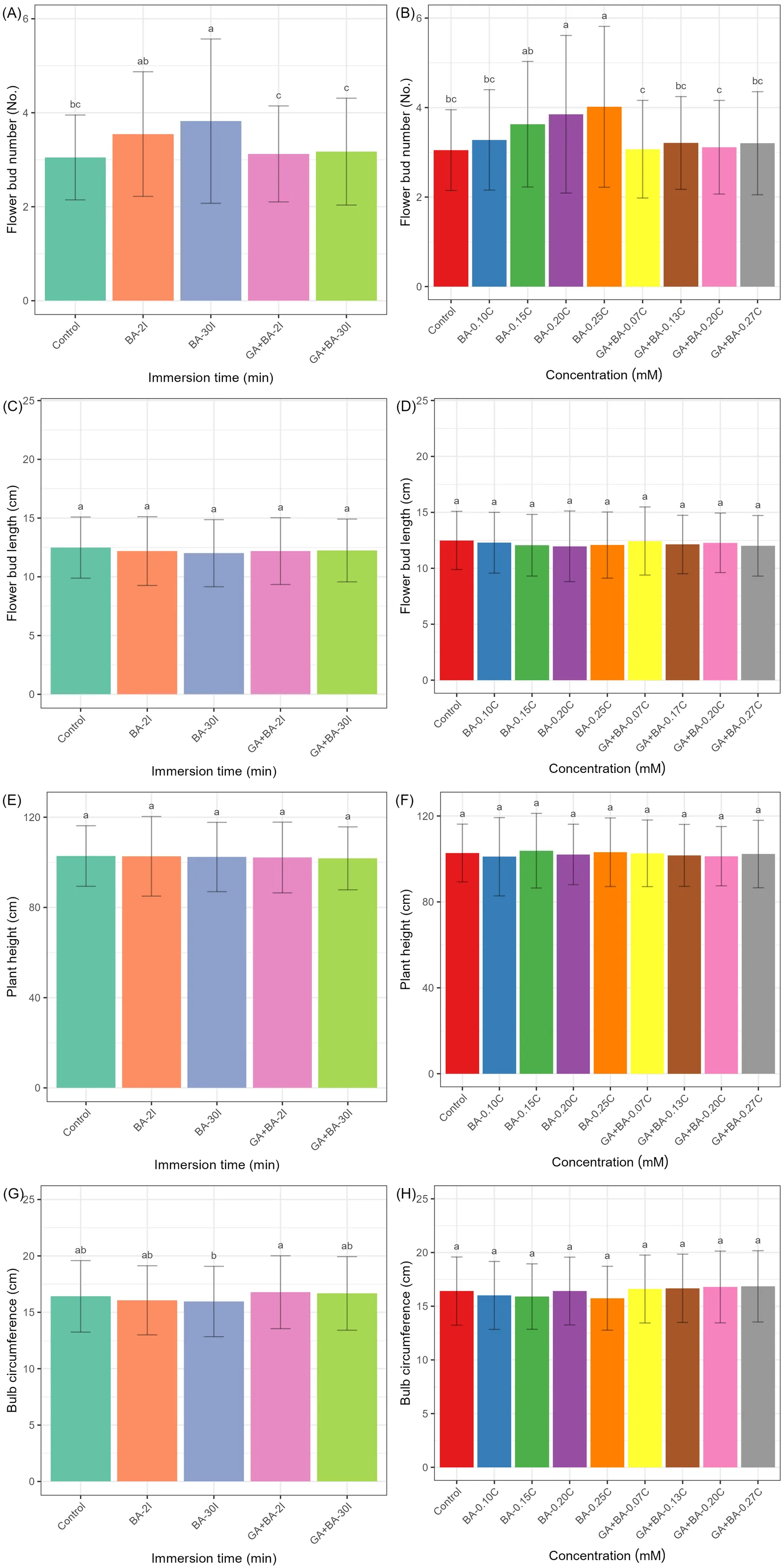
Effects of PGR treatments on quality-related traits of small- and medium-sized lily bulbs under different immersion times (2 or 30 min) **(A, C, E, G)** and concentrations (BA: 0, 0.10, 0.15, 0.20, and 0.25 mM; GA+BA: 0, 0.07, 0.13, 0.20, and 0.27 mM) **(B, D, F, H)**. Immersion time treatments are labeled by PGR type and immersion duration, followed by the letter I. Concentration treatments are labeled by PGR type and concentration level, followed by the letter C. The vertical bars represent 95% confidence intervals for the given experimental conditions, and the lowercase letters (a–c) indicate significant differences among means, with a, b, and c ordered from the longest (largest) to the shortest (smallest) average for each trait. GA, gibberellin; BA, benzylaminopurine; PGR, plant growth regulators.

When small- and medium-sized bulbs were immersed in BA for 30 min, flower bud number (3.82) was significantly higher than that in the control (3.05; *p* = 0.0003) (**Figure 1A**). The highest flower bud number (4.02) was observed when the BA concentration was 0.25 mM (**Figure 1B**). At a BA concentration of 0.20 mM, the flower bud number was 3.85, which was not significantly different from that in the 0.25 mM BA group. Both the 0.20 mM and 0.25 mM BA treatments produced significantly more flower buds than the untreated control (3.05; *p* < 0.05), whereas the 0.10 and 0.15 mM treatments did not differ from the control. Thus, 0.20 mM was the lowest BA concentration at which flower bud number significantly exceeded the control (**Figure 1B**).

When small- and medium-sized lily bulbs were treated with GA+BA, flower bud number was unaffected by immersion time or treatment concentration. Therefore, although the PGR sold under the brand name Pomina (GA+BA) is widely used in South Korea to increase flower bud number, this study demonstrated that the treatment is ineffective in improving this trait in small- and medium-sized lily bulbs.

Overall, immersing bulbs in 0.20 mM BA for 30 min can increase flower bud number and produce high-quality cut flowers. However, aside from flower bud number, other quality-related traits (flower bud length, **Figures 1C, D**; plant height, **Figures 1E, F**; bulb circumference, **Figures 1G, H**) did not differ significantly among treatments.

### 3.2 Effects of PGR treatment by cultivar

**Figure 2:**
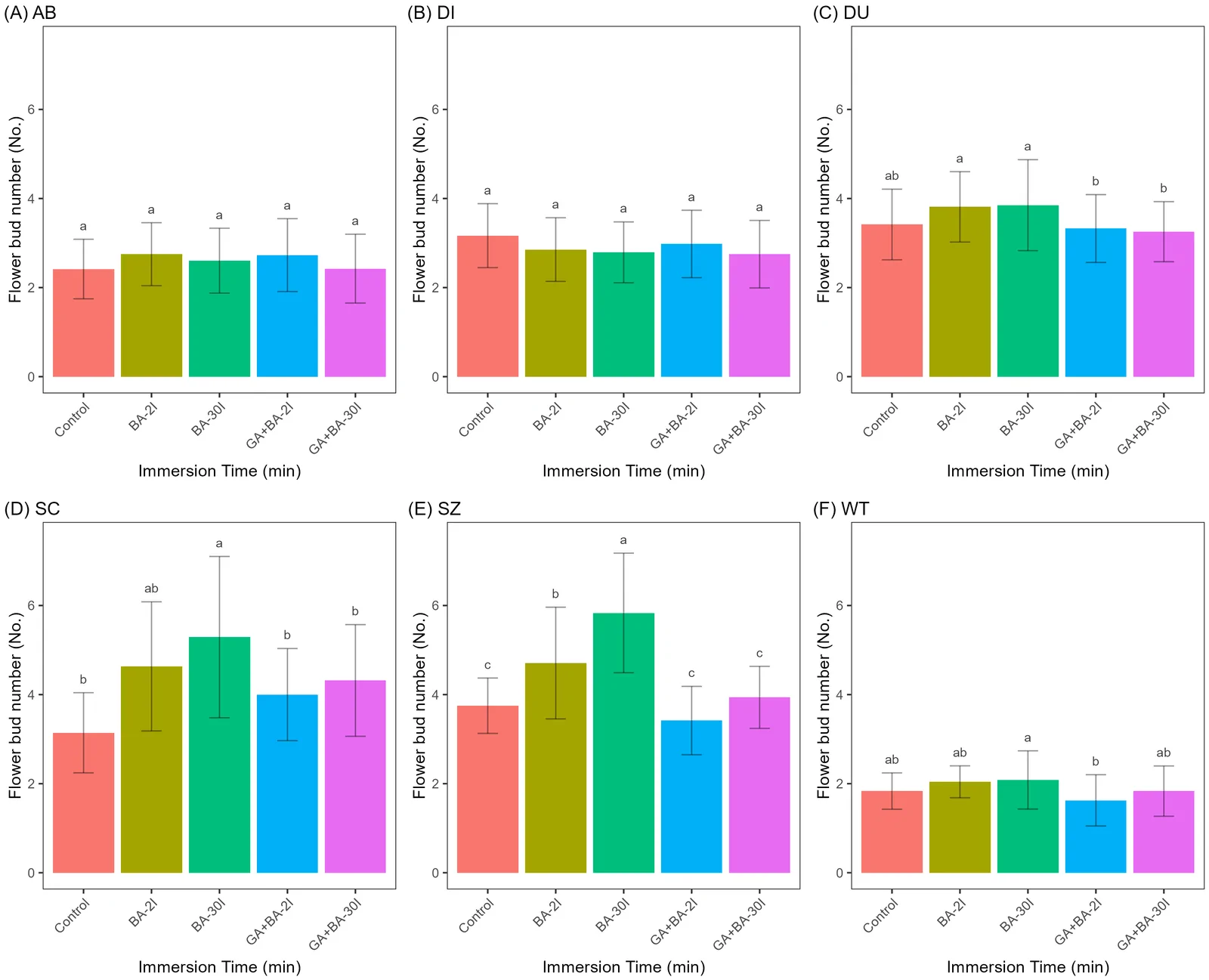
Effects of PGR immersion time on flower bud number in different cultivars. **(A)** Aubisque (AB); **(B)** Difference (DI); **(C)** Ducati (DU); **(D)** Spring City (SC); **(E)** Sweet Zanica (SZ); **(F)** Woori Tower (WT). Different letters above the bars indicate significant differences among treatments within each cultivar (*p* < 0.05). Immersion time treatments are labeled by PGR type and immersion duration, followed by the letter I. GA, gibberellin; BA, benzylaminopurine; PGR, plant growth regulators.

The effects of PGR treatment on flower bud number in small- and medium-sized lily bulbs were analyzed across different cultivars (**Figure 2**, **Supplementary Figures 1**, **2**). Of the six cultivars, only SC and SZ showed an increase in flower bud number when treated with BA. When these two cultivars were immersed in BA for 30 min, the flower bud number was 5.29 for SC and 5.83 for SZ (**Figure 2**); the highest flower bud numbers were observed following treatment with 0.25 mM BA, at 6.25 for SC and 5.91 for SZ (**Supplementary Figure 1**).

In DU, flower bud number did not differ between BA-treated and control plants (*p* = 0.996) (**Figure 2C**). DU exhibited an average of 4.12 flower buds after 30 min immersion in 0.25 mM BA, which did not satisfy the premium criterion of ≥5 (**Supplementary Figure 1**). AB, DI, and WT did not show increased flower bud numbers after any of the PGR treatments. Compared with the control group, AB showed an increased number of flower buds after BA-2I and BA-30I treatment, at 2.75 and 2.60 buds, respectively; however, this was not significant. DI trended toward decreased flower bud number after BA-2I and BA-30I treatment, at 2.85 and 2.79 buds, respectively, compared with that in the control group (3.17). WT showed the lowest number of flower buds across all treatment groups. The control produced 1.83 flower buds, and PGR treatments yielded similar values (1.63–2.08), with no significant differences among treatments.

When plants were treated with GA+BA, even at different immersion times and concentrations, none of the cultivars showed an increase in the number of flower buds (**Figure 2**, **Supplementary Figure 2**). SC and SZ showed a trend toward increasing flower bud numbers with longer GA+BA immersion times, but this was not significant.

Notably, responsiveness to BA did not correspond to lineage. Of the two LA hybrids, SZ responded and DU did not; of the two Oriental cultivars, SC responded and AB did not. Neither *L. longiflorum* cultivar responded. Responsiveness therefore segregated within lineages rather than between them (**Table 1**).

**Figure 3:**
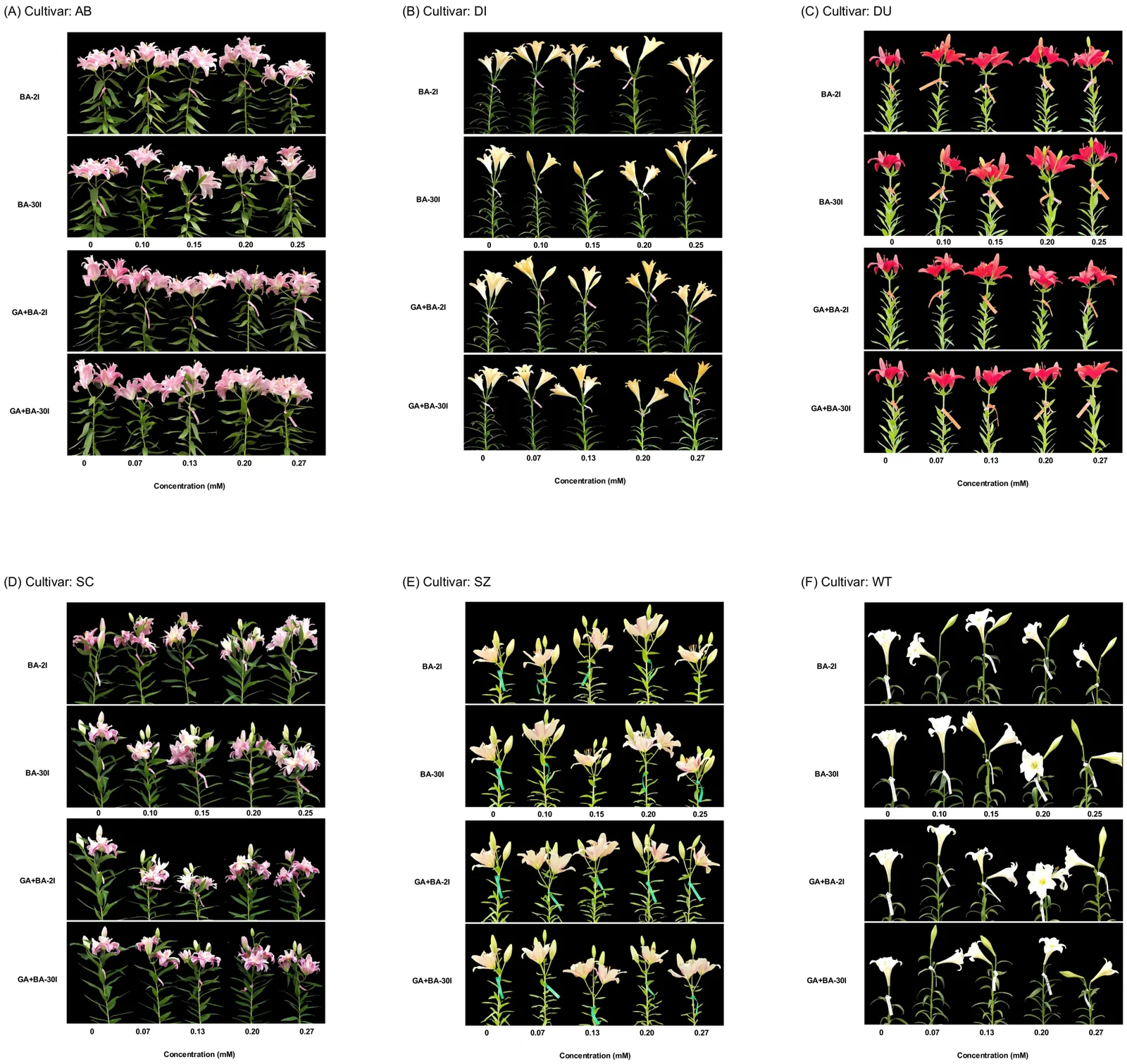
Flower morphology of lily cultivars under PGR treatments at different concentrations (BA: 0, 0.10, 0.15, 0.20, 0.25 mM; GA+BA: 0, 0.07, 0.13, 0.20, 0.27 mM) and immersion times (2 or 30 min). **(A)** Aubisque (AB); **(B)** Difference (DI); **(C)** Ducati (DU); **(D)** Spring City (SC); **(E)** Sweet Zanica (SZ); **(F)** Woori Tower (WT). Immersion time treatments are labeled by PGR type and immersion duration, followed by the letter I. Concentration treatments are labeled by PGR type and concentration level, followed by the letter C. GA, gibberellin; BA, benzylaminopurine; PGR, plant growth regulators.

Morphological changes in the six cultivars when treated with PGRs were examined from photographs (**Figure 3**). SC and SZ had more flower buds than the control group after 30 min of immersion in 0.20 mM BA, confirming the prior findings. Meanwhile, for AB, DI, DU, and WT, the differences in the number of flower buds, compared with those in the control, were not significant for any BA treatment groups, and none of the cultivars were affected by the GA+BA treatment. These observations are consistent with the treatment effects shown in **Figure 2**. An objective comparison of plant height using photographs indicated that plant height decreased in SC and SZ following treatment with 0.25 mM BA-30I.

### 3.3 Frequency distribution of grades of quality-related traits

**Figure 4:**
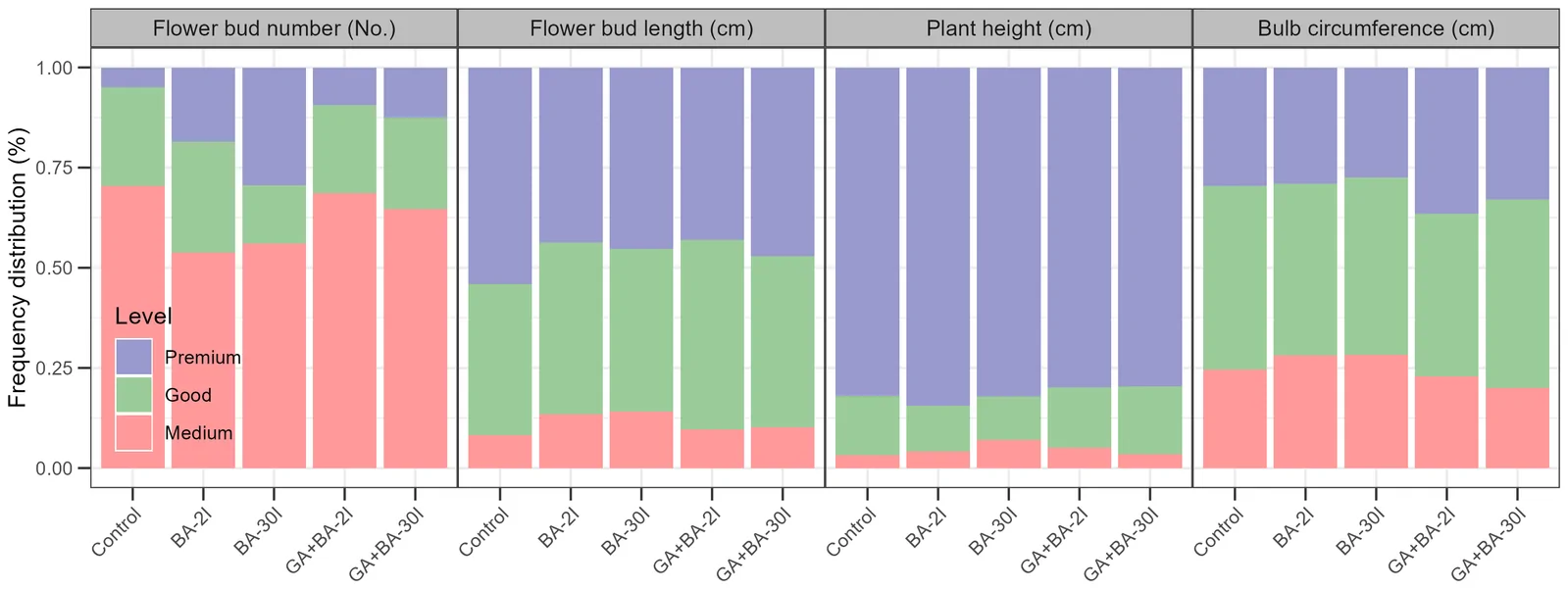
Classification of quality grades based on the Lily Export Standards for Japan. Flower bud number: premium (≥5 buds), good (4 buds), and medium (<4 buds); flower bud length: premium (≥12 cm), good (9.5–11.9 cm), and medium (<9.5 cm); plant height: premium (≥90 cm), good (80.1–89.9 cm), and medium (≤80 cm); bulb circumference: premium (>18 cm), good (14.0–18.0 cm), and medium (<14 cm). Immersion time treatments are labeled by PGR type (BA or GA+BA) and immersion duration (2 or 30 min), followed by the letter I. The y-axis represents the frequency distribution of quality grades. GA, gibberellin; BA, benzylaminopurine; PGR, plant growth regulators.

We investigated the effects of PGR immersion time and concentration, as well as cultivar, on the distribution of grades for export quality-related traits (**Figure 4**, **Supplementary Figures 3**, **4**).

The grading criteria for flower bud number were defined as premium (≥5 buds), medium (<4 buds), and good (intermediate values). The images consistently confirmed the treatment effects illustrated in **Figure 4**. The frequency distribution of flower bud numbers in the untreated sample demonstrated that the proportions of premium (5%) and good (25%) grades were much lower than that of medium grade (70%) (**Figure 4**). However, when the bulbs were immersed in BA for 30 min (BA-30I), the proportion of premium grades rose from 5% to 29%, while the good grade fell from 25% to 15%, so that the combined high-quality proportion increased from 30% to 44%. Although the proportion of high-quality grades (premium + good) was higher for BA-2I (46%) than that for BA-30I (44%), the proportion of premium grades for flower bud number was markedly higher in the BA-30I group.

Flower bud length and plant height were not affected by PGR immersion time. Meanwhile, bulb circumference (**Figure 4**) showed an increase in the proportion of high-quality grades (76%; premium 36% + good 40%) in GA+BA-2I, compared with that in the control (high-quality 74%). Notably, the GA+BA-2I group showed the highest proportion of premium grades.

The effects of PGR concentration on quality-related traits were also examined (**Supplementary Figure 3**). For flower bud number, the proportion of high-quality grades (48%; premium 27% + good 21%) in the BA-0.20C group was higher than that in the control group (high-quality 28%). In the BA-0.25C group, the proportion of high-quality grades was 45%, which was lower than that in BA-0.20C, suggesting 0.20 mM as the most effective BA concentration for increasing flower bud number in small- and medium-sized lily bulbs. Nevertheless, flower bud length and plant height were not affected by PGRs, and the proportion of high-quality grades was comparable to or lower than that in the control group. Bulb circumference showed 80% high-quality grades following BA-0.20C treatment, which was higher than that in the control group (74%). Treatment with GA+BA also showed a trend-level increase, compared with that in the control group.

Quality frequency was also analyzed for each cultivar, and all four traits showed cultivar-specific responses (**Supplementary Figure 4**). Of the six cultivars, the most suitable cultivars for export were SC and SZ. SC showed the highest proportion of high-quality grades, at 78%, followed by SZ at 74%. DU showed the third-highest proportion of high-quality grades (41%), which was insufficient for export standards. AB, DI, and WT were more suited for the production of cut flowers for domestic use rather than export. In particular, WT did not produce cut flowers with 4 or more flower buds and also showed the lowest proportion of premium grades for plant height (≥90 cm).

SC showed the lowest proportion of high-quality grades for bulb circumference after harvesting (high-quality 28%; premium 0.6% + good 27.4%), suggesting that it would be disadvantageous for the production of cut flowers in subsequent years, compared with the other cultivars. For SZ, the proportion of high-quality grades for bulb circumference after harvesting was 89% (premium 34% + good 55%), and because this is also effective for increasing the number of flower buds, SZ was determined to be a promising cultivar for producing cut flowers in the subsequent year.

### 3.4 PCA

**Figure 5:**
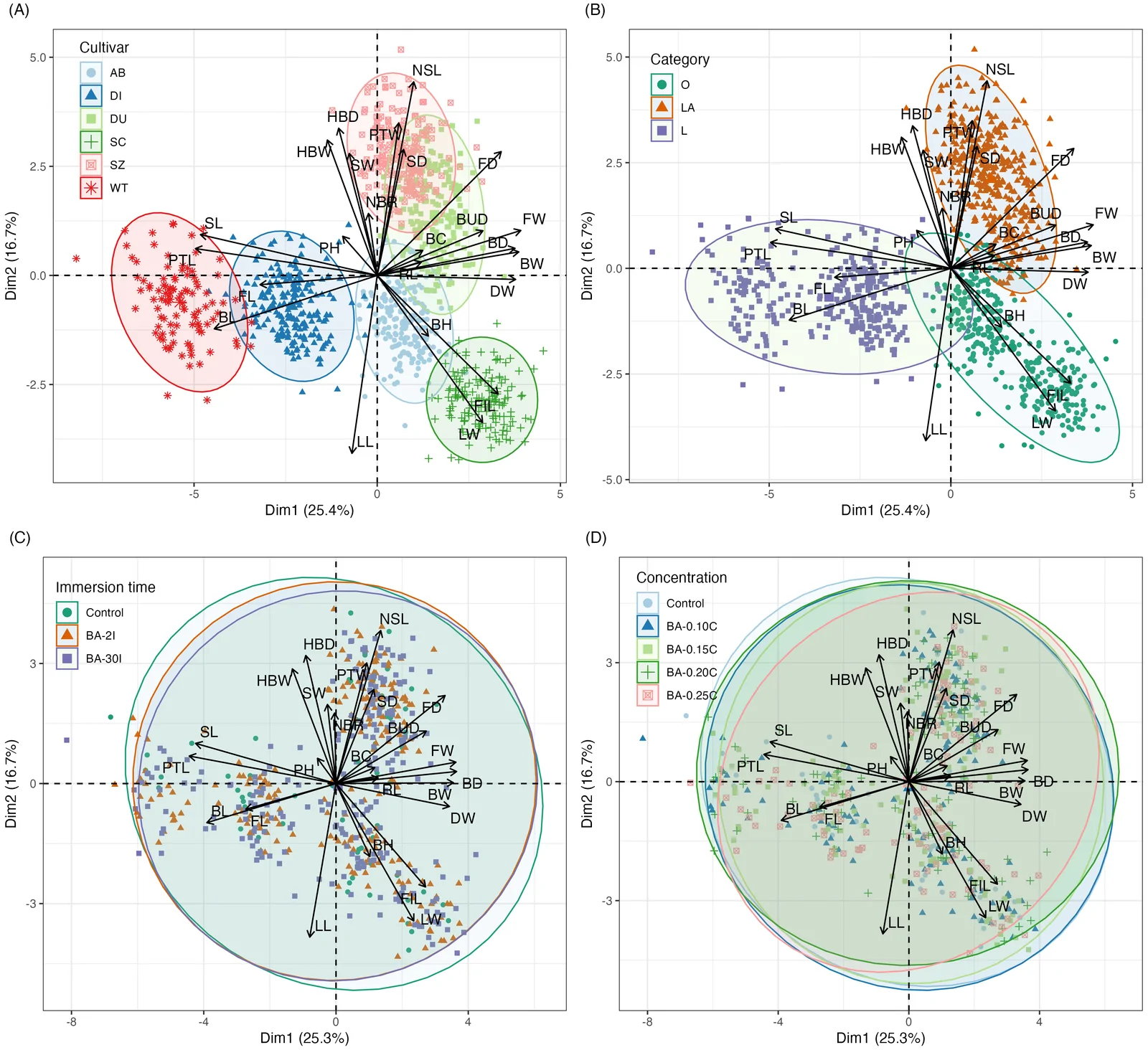
Principal component analysis (PCA) biplots showing the PCA scores of explanatory variables as vectors (black arrows) and individuals (each point). **(A)** Each class represents a lily cultivar: Aubisque (AB, light blue circles), Difference (DI, blue triangles), Ducati (DU, light green squares), Spring City (SC, green crosses), Sweet Zanica (SZ, pink crossed squares), and Woori Tower (WT, red asterisks). **(B)** Each class represents a lily type: Oriental (O, green circles), LA hybrid (LA, orange triangles), and Longiflorum (L, purple squares). **(C)** Each class represents immersion time: Control (green circles), BA-2I (orange triangles), and BA-30I (purple squares). Immersion time treatments are labeled by PGR type and immersion duration, followed by the letter I. **(D)** Each class represents concentration treatments of BA: Control (light blue circles), BA-0.10C (blue triangles), BA-0.15C (light green squares), BA-0.20C (green crosses), and BA-0.25C (pink crossed squares). Concentration treatments are labeled by PGR type and concentration level, followed by the letter C. BA, benzylaminopurine; PGR, plant growth regulators.

PCA was performed on all the investigated traits for each cultivar (**Figure 5A**), lily type (**Figure 5B**), PGR immersion time (**Figure 5C**), and PGR concentration (**Figure 5D**). Each cultivar was separated according to its cultivar-specific traits. Cultivars positioned in the direction of the flower bud number vector, the most important quality-related trait, included SZ, SC, and DU. Conversely, AB, DI, and WT were positioned in the direction opposite to the flower bud number vector, indicating that these cultivars were characterized by low flower bud numbers. Flower bud number had a relatively strong correlation with flowering width (*r* = 0.371), fresh weight (*r* = 0.369), and number of stem leaves (*r* = 0.337) (**Supplementary Figures 5**, **6**). Consistent with these results, SZ and DU showed the largest bulb circumferences, with SC being the smallest. Bulb circumference was strongly correlated with bulb weight (*r* = 0.933; **Supplementary Figure 5**). Analysis by lily type revealed that, of the LA hybrids (SZ and DU) and Oriental lines (AB and SC), SZ showed the highest number of flower buds. Meanwhile, the *L. longiflorum* lines (DI and WT) had a low flower bud number, indicating characteristics unsuitable for export. Flower bud length was specifically high for the *L. longiflorum* lines. Bulb circumference after harvest tended to be higher for LA lines, such as SZ and DU. When the distribution of samples was examined by PGR immersion time, the three ellipses (control and two treatment groups) almost overlapped. However, the ellipses for BA-2I and BA-30I were slightly larger than that of the control in the direction of the flower bud number vector, demonstrating that BA immersion effectively increased the number of flower buds. Similarly, in the analysis by PGR concentration, the ellipses for 0.20 mM BA and 0.25 mM BA were larger than that of the control in the direction of the flower bud number vector.

Bulb circumference after harvest was the highest for the control group ellipse, suggesting a possible reduction in bulb circumference after harvest under BA treatment. The separation from the control was greater at the longer immersion time (BA-30I) and the higher concentration (BA-0.25C). The flower bud length and plant height traits showed similar distributions to those of the control group, suggesting that these traits were not affected by BA treatment.

### 3.5 Regression model for estimating quality-related traits

**Figure 6:**
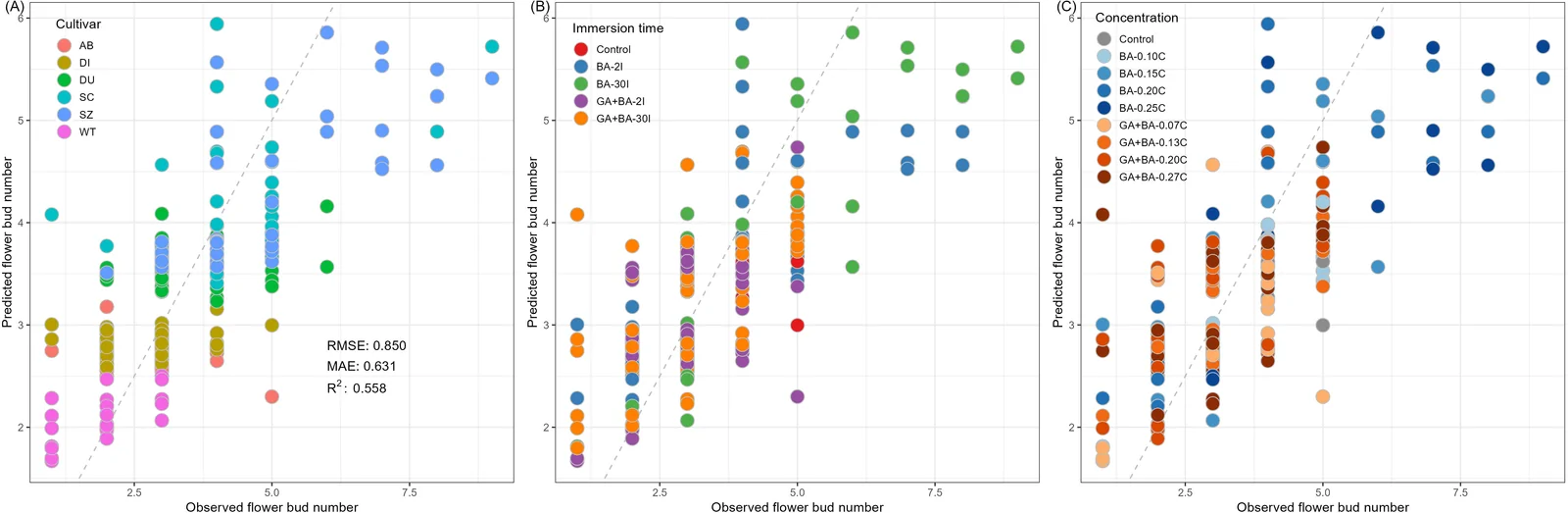
Scatter plots of observed vs. predicted number of flower buds based on datasets for cultivar **(A)**, immersion time **(B)**, and concentration **(C)** of PGR types. Each point represents an individual sample. The 1:1 slope is represented by a gray dotted line. Predicted values span a narrower range than observed values, reflecting the shrinkage towards the mean characteristic of regularised ensemble regression. GA, gibberellin; BA, benzylaminopurine; PGR, plant growth regulator; AB, Aubisque; DI, Difference; DU, Ducati; SC, Spring City; SZ, Sweet Zanica; WT, Woori Tower; RMSE, root mean square error; MAE, mean absolute error.

**Figure 7:**
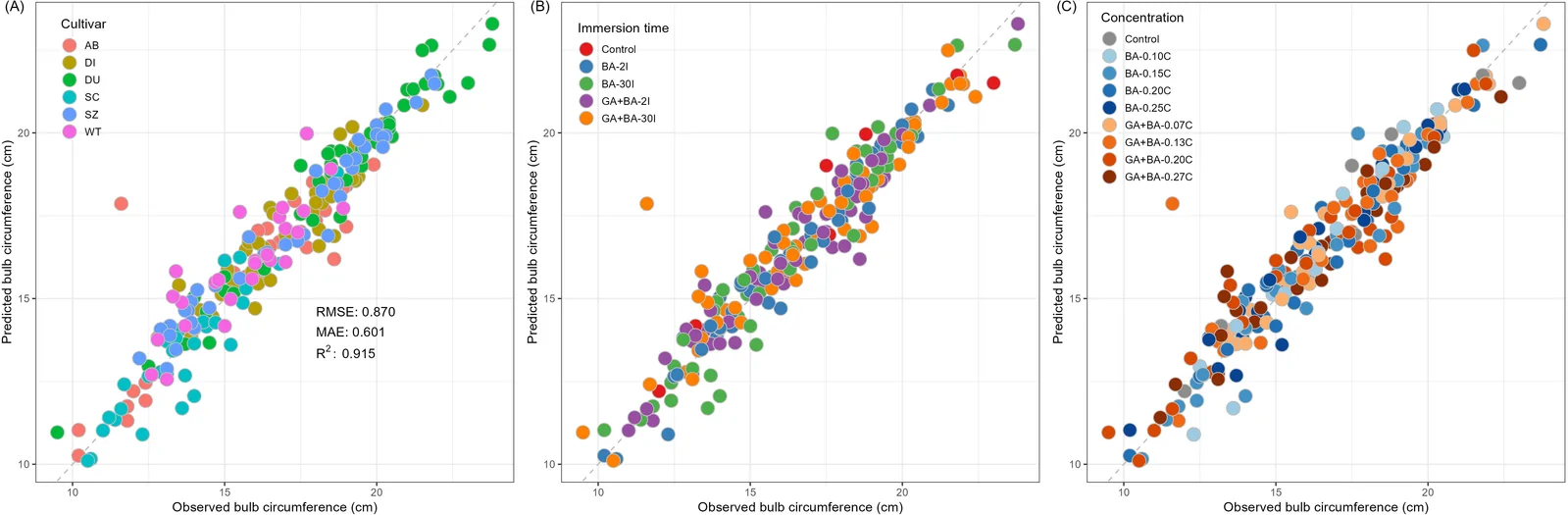
Scatter plots of observed vs. predicted bulb circumference based on datasets for cultivar **(A)**, immersion time **(B)**, and concentration **(C)** of PGR types. Each point represents an individual sample. The 1:1 slope is represented by a gray dotted line. GA, gibberellin; BA, benzylaminopurine; PGR, plant growth regulator; AB, Aubisque; DI, Difference; DU, Ducati; SC, Spring City; SZ, Sweet Zanica; WT, Woori Tower; RMSE, root mean square error; MAE, mean absolute error.

Based on the analyzed traits, machine learning models were developed to predict quality-related traits (**Figures 6**, **7**, **Supplementary Figures 7**-**9**). In the model for predicting flower bud number, traits such as the number of stem leaves were important for model performance (**Figure 6**, **Supplementary Figure 9**). In terms of performance, the flower bud number prediction model showed a root mean square error of 0.850, a mean absolute error of 0.631, and an *R*² of 0.558. The model-predicted values were analyzed according to cultivar type (**Figure 6A**), immersion time (**Figure 6B**), and treatment concentration (**Figure 6C**), and the findings were consistent with the aforementioned results. When the model was tasked with predicting flower bud numbers ≥5, among the six cultivars, SC and SZ exhibited the highest flower bud numbers. Similarly, samples immersed in BA for 30 min and treated with 0.25 mM BA also displayed the highest flower bud numbers. In contrast, GA+BA treatment did not increase flower bud numbers.

Evaluated against the operational criterion, namely whether a plant reaches the premium standard of at least five flower buds, the model achieved an area under the receiver operating characteristic curve (AUC) of 0.881 (95% CI 0.857–0.906), with a sensitivity of 89.7% and a specificity of 71.3% at the cut-off maximizing Youden’s J (**Supplementary Figure 10**). The predicted values in **Figure 6** span a narrower range than the observed values, reflecting the shrinkage towards the mean that is characteristic of regularized ensemble regression; the optimal cut-off on the predicted scale is consequently below five, and applying a literal threshold of five to the predicted values would substantially reduce sensitivity. The moderate coefficient of determination therefore understates the usefulness of the model for the categorical decision for which it is intended, although it remains insufficient for precise prediction of individual bud counts.

A second model restricted to pre-planting information (cultivar, PGR type, concentration, immersion time and bulb attributes measured before planting) achieved a coefficient of determination of *R*² = 0.546, with a root mean square error of 0.901 buds. When evaluated as a classifier against the same premium threshold (≥5 flower buds), this model achieved an AUC of 0.886 (95% CI 0.862–0.910), essentially identical to that of the full trait set (0.881, 95% CI 0.857–0.906; **Supplementary Figure 10**). Thus, although its R² was lower, the pre-planting model discriminates premium-grade lots as well as the full model; because it relies only on information available before crop establishment, it can support lot-level decisions on whether BA immersion is justified at the point at which that decision is taken.

Leave-one-cultivar-out cross-validation, in which the model was trained on five cultivars and evaluated on the sixth, yielded coefficients of determination ranging from -6.379 to -0.484. Performance on held-out cultivars was substantially lower than under standard cross-validation, indicating that the relationship between growth traits and flower bud number is cultivar-specific and does not extrapolate reliably to cultivars absent from the training data. This provides quantitative support for the conclusion that the determinants of vegetative growth traits and flower bud number in lily are largely cultivar-specific. Cultivar dependence, previously inferred from the treatment responses alone, could thereby be demonstrated quantitatively rather than descriptively.

The model for estimating bulb circumference after harvest showed strong prediction performance (*R*² = 0.915; **Figure 7**). SZ, DU, and DI were characterized by a larger bulb circumference after harvesting, compared with other cultivars, whereas SC and AB had a lower bulb weight. PGR treatment (immersion time and concentration) did not increase the bulb circumference. Bulb circumference after harvest was strongly correlated with post-harvest bulb weight (**Supplementary Figure 5**), which markedly contributed to the strong prediction performance of this model. We note that this accuracy is largely attributable to the inclusion of post-harvest bulb weight, which is measured on the same organ at the same time as bulb circumference and is correlated with it at *r* = 0.933 (**Supplementary Figure 5**); the dominance of this variable is evident in the variable importance analysis (**Supplementary Figure 9B**). The model should therefore be understood as confirming the internal consistency of the bulb measurements rather than as demonstrating predictive capability, and the interpretive weight of the modeling component rests on the flower bud number model and on the trait ranking derived from it.

In the model for predicting flower bud length, cultivar emerged as the most important feature (**Supplementary Figure 7**). Specifically, flower bud length was the most affected by cultivar and was not sensitive to environmental effects such as PGR treatment (immersion time or concentration). DI exhibited the longest flower buds among the six cultivars, with DU being the shortest.

Similar to the flower bud length model, the plant height prediction model was strongly affected by cultivar or genotype (**Supplementary Figure 8**). Plant height was not affected by PGR treatment, indicating that PGR treatment was ineffective at increasing plant height. Consistent with the results for flower bud length, DI had the greatest plant height, whereas WT had the shortest plant height. In addition to flower bud number, plant height in WT was below the standards required for export, reaffirming the unsuitability of this cultivar for export.

### 3.6 Establishment of optimal cultivation conditions to maximize flower bud number using RSM analysis

**Figure 8:**
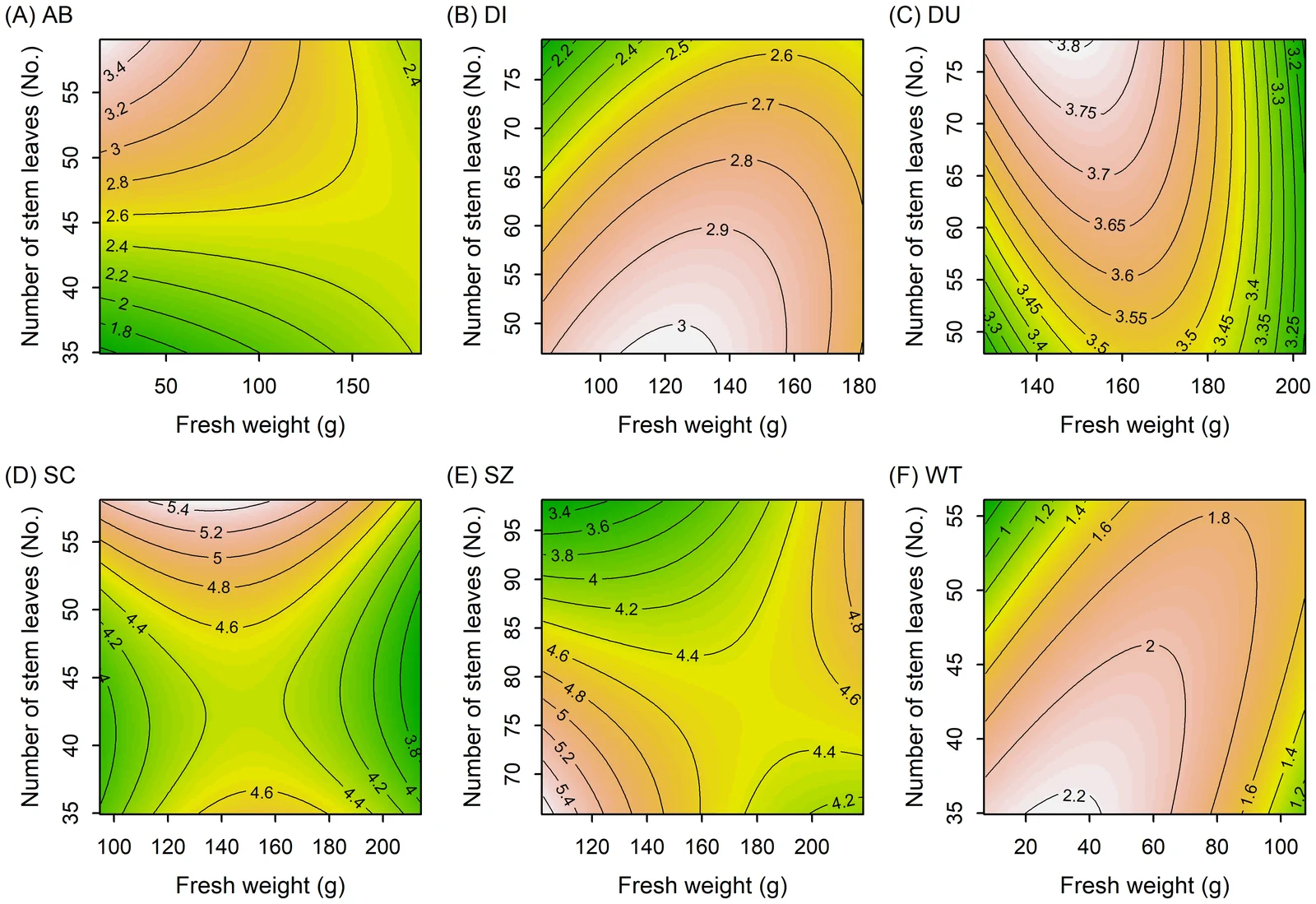
Estimated response surface of flower bud number (brighter colors indicate higher values) using shoot fresh weight (x-axis) and the number of stem leaves (y-axis) for each cultivar. **(A)** Aubisque (AB); **(B)** Difference (DI); **(C)** Ducati (DU); **(D)** Spring City (SC); **(E)** Sweet Zanica (SZ); **(F)** Woori Tower (WT). Contour labels give the expected number of flower buds. Surfaces are shown only within the range of the observed data.

To establish a cultivation method and maximize the number of flower buds from small- and medium-sized lily bulbs, RSM analysis was applied (**Figure 8**). First, traits that are both strongly correlated with flower bud number and can be artificially controlled by growers were selected (**Supplementary Figures 5**, **6**). Compared with other traits, fresh weight (*r* = 0.37) and number of stem leaves (*r* = 0.34) showed relatively stronger correlations with flower bud number. The expected flower bud number was visualized as a contour surface over shoot fresh weight and number of stem leaves for each cultivar, and the surfaces differed markedly among cultivars in both the level attained and the location of the optimum.

Only SC and SZ, the two cultivars that responded to BA treatment, attained the premium standard of at least five flower buds at any combination of the two traits within the range of the observed data. In SC the expected value ranged from 3.6 to 5.5, with the maximum occurring at an intermediate shoot fresh weight of approximately 135 g combined with the highest stem leaf numbers recorded for this cultivar, approximately 58. In SZ the expected value ranged from 3.3 to 5.7, with the maximum occurring at the lower end of both variables, approximately 102 g fresh weight and 66 stem leaves.

The remaining four cultivars did not attain five flower buds anywhere within the observed range, and none of them attained the good standard of four buds either. AB reached a maximum of 3.6 at low fresh weight, approximately 50 g, combined with a high stem leaf number of approximately 59, so that the expected bud number declined as fresh weight increased. DU reached 3.8 at an intermediate fresh weight of approximately 148 g and the highest stem leaf number recorded for this cultivar, approximately 78. DI reached only 3.0, at an intermediate fresh weight of approximately 121 g and the lowest stem leaf number, approximately 47. WT reached 2.2 at the lower end of both variables and remained below three buds across the whole surface.

The direction of the association with stem leaf number therefore differed among cultivars, being positive in AB, DU and SC and negative in DI, SZ and WT. This heterogeneity indicates that a single set of target growth conditions cannot be recommended across cultivars, and that the growth state associated with maximal flower bud number must be established separately for each.

The response surfaces are presented as cultivar-specific descriptions of the growth state associated with maximal flower bud number, that is, as in-season growth targets, rather than as pre-planting decision rules. Both predictors are management-modifiable, being determined by bulb lot quality, planting density, fertigation and the greenhouse environment, and both can be assessed before anthesis, so that lots can be allocated to export or domestic channels before harvest.

To render the response surfaces usable in the field, **Table 3** summarizes for each cultivar the combinations of shoot fresh weight and stem leaf number associated with the premium standard of at least five flower buds and with the good standard of at least four. Neither standard was attained at any combination within the observed data range in AB, DI, DU or WT. In SC and SZ the premium standard was attained over a restricted part of the surface, corresponding to 10% and 6% of the area examined respectively, whereas the good standard was attained over most of it. The tabulated ranges are bounding intervals of regions that are not rectangular, and should therefore be read together with the contours in **Figure 8**; they should not be extrapolated beyond the range of the observed data.

**Table 3:** Cultivar-specific ranges of shoot fresh weight and stem leaf number associated with the premium (≥5 buds) and good (≥4 buds) standards, estimated from the second-order response surfaces in **Figure 8** within the range of the observed data.

Cultivar	Target	Fresh weight (g)	Stem leaves (no.)	Maximum predicted buds
SC	≥5	95–186	54–58	5.5
SC	≥4	95–214	35–58	5.5
SZ	≥5	102–133	66–78	5.7
SZ	≥4	102–218	66–98	5.7
AB	≥5	Not attained	—	3.6
DI	≥5	Not attained	—	3.0
DU	≥5	Not attained	—	3.8
WT	≥5	Not attained	—	2.2

Ranges are bounding intervals within the range of the observed data and should be interpreted together with the contours in **Figure 8**, since the region of attainment is not rectangular in most cultivars. AB, Aubisque; DI, Difference; DU, Ducati; SC, Spring City; SZ, Sweet Zanica; WT, Woori Tower.

## 4 Discussion

### 4.1 Effects of PGR type, concentration and immersion time on flower bud number

In this study, we demonstrated that PGR treatment influences flower bud number in small- and medium-sized lily bulbs. First, we analyzed the effects of BA and GA+BA concentrations and immersion times on flower bud number in six lily cultivars. BA-30I considerably increased flower bud number, with SC and SZ cultivars displaying the highest averages. Among all cultivars, SZ showed the greatest increase in flower bud number and the highest potential for bulb size improvement after harvest. Although higher BA concentrations were predicted to increase the flower bud numbers in the present study, the values at 0.20 and 0.25 mM were similar, suggesting that the effect of BA treatment was already saturated at 0.20 mM. A previous study on PGR treatment methods identified hormone concentration and treatment time as the most important factors (Sajjad et al., 2017). Similarly, in our study, a BA treatment duration of 30 min was more effective than 2 min in increasing flower bud number. These findings suggest that treatment for 30 min is sufficient for BA absorption and the induction of a physiological response.

Although treatment with BA alone clearly increased flower bud number, treatment with GA+BA showed no time- or concentration-dependent changes. The two formulations were applied over comparable ranges of total PGR molarity (BA, 0.10–0.25 mM; GA+BA, 0.07–0.27 mM), on the expectation that mixed formulations would be effective at lower total concentrations owing to synergistic interactions between GA and cytokinin components. Because the mixed formulation delivers the two active ingredients in a 1:1 ratio by mass, however, the cytokinin component was necessarily lower than in the corresponding BA-alone treatments: the highest GA+BA concentration tested delivered 0.161 mM BA (**Table 2**), below the 0.20 mM at which BA alone first became effective. GA+BA treatment did not significantly increase flower bud number at any concentration. In the present experiment the mixed formulation altered none of the four export quality traits, including plant height (**Figures 1E, F**). Gibberellin is generally reported to promote stem elongation in ornamental species (Zhang et al., 2022), and the absence of such an effect here is consistent with the low gibberellin dose delivered by the formulation at the concentrations tested (0.028–0.109 mM; **Table 2**) and with the pre-planting route of application.

The effects of PGR treatment were assessed by examining the distribution of quality grades. By analyzing the distribution of premium, good, and medium grades according to standards for export to Japan, we verified the actual commercial value. Although the ratio of high-quality grades was only 30% (premium 5% + good 25%) in the control group, 30-min immersion in BA increased the proportion of high-quality grades to 44% (premium 29% + good 15%). This indicates that, by increasing flower bud number, BA treatment increases the proportion of high-quality plants that satisfy standards for export to Japan. A concentration of 0.20 mM BA was the most efficient in improving the quality grade. Although a concentration of 0.25 mM resulted in the highest number of flower buds, the distribution of quality grades showed that 0.20 mM actually resulted in a higher proportion of high-quality grades (48%) than 0.25 mM (45%). Therefore, considering the economics and production of high-quality cut flowers, 0.20 mM was determined to be the optimal concentration, and the results indicate that further increasing the concentration does not necessarily improve quality.

### 4.2 Cultivar-dependent responses and candidate hormonal mechanisms

The interpretations offered in this subsection are hypotheses derived from the literature. The present study measured phenotypic responses only; endogenous hormone concentrations, hormone receptor and signaling gene expression, and carbohydrate pools were not quantified. In addition, the mechanistic literature we draw on concerns bulbil initiation, the formation of aerial axillary propagules, rather than floral bud initiation. The two processes share a dependence on axillary meristem activation and on cytokinin signaling, and Shu et al. (2024) identify the shared genetic basis of bulbil and flower formation as an open question; the mechanisms discussed below are therefore presented as analogies to be tested in lily floral development, not as established explanations of our results.

Within this framework, initiation of meristem-derived organs in bulbous species is governed by an interaction among cytokinin, gibberellin and auxin (Shu et al., 2024). Cytokinin acts positively on initiation: in *Lilium lancifolium*, cytokinins promote bulbil initiation in the leaf axils, and exogenous 6-benzylaminopurine induces bulbil initiation by promoting cell proliferation. Key cytokinin biosynthesis and activation genes (*LOG*, *CYP735A1*) and the signal transduction histidine kinase *AHK2* are upregulated during initiation, while the degradative *CKX* is co-regulated, indicating that initiation is associated with active cytokinin turnover rather than simple accumulation. Auxin appears to act indirectly and in a stage-dependent manner, promoting initiation but subsequently restraining growth, and the ratio of zeatin to indole-3-acetic acid rather than the concentration of either alone has been identified as the determinant of initiation in several species. This framework provides a plausible basis for the response we observed to a 30-min immersion in 0.20 mM BA, in which flower bud number increased in responsive cultivars while no other quality trait was affected.

Two non-exclusive explanations can be offered for the absence of any effect of GA+BA on flower bud number, of which the first is the more parsimonious. The first is dosimetric. Because the mixed formulation contains gibberellin and BA in a 1:1 ratio by mass, the cytokinin delivered was 0.042, 0.078, 0.119 and 0.161 mM across the four concentrations tested (**Table 2**). None of these reaches 0.20 mM, the lowest concentration at which BA applied alone produced a significant increase in flower bud number, and the highest of them lies between 0.15 mM, which was ineffective when BA was applied alone, and 0.20 mM, which was effective. The absence of a response to GA+BA is therefore consistent with the cytokinin dose delivered, without any need to invoke an interaction between the two hormones. We regard this as the primary explanation and note that it constitutes a limitation of the design rather than a biological finding: the mixed formulation was never tested at a cytokinin dose known to be effective in this study.

The second explanation, which our data cannot exclude but do not require, is antagonism. GA acts predominantly on the expansion of organs that have already been initiated rather than on initiation itself, and in bulbous species exogenous gibberellin reduces the number of meristem-derived organs while increasing the size of individual organs; inhibition of gibberellin biosynthesis has the opposite effect (Shu et al., 2024). If a comparable relationship governs floral primordium initiation in lily, the gibberellin component of the mixed formulation could offset the initiation-promoting effect of the cytokinin component. The direction of our other results is consistent with such a relationship: BA alone increased bud number but reduced post-harvest bulb circumference, whereas GA+BA left bud number unchanged and was associated with a trend towards larger bulb circumference. The two explanations can be separated by a dose-matched factorial experiment in which BA is held at 0.20 mM and gibberellin is added at a graded series of concentrations; a monotonic decline in bud number with increasing gibberellin would support antagonism, whereas equivalence with BA alone would confirm that the present result reflects cytokinin dose alone. Until such an experiment is performed, the conclusion that can be drawn from these data is that the mixed formulation, applied according to the manufacturer’s concentration range, does not increase flower bud number, and not that gibberellin antagonizes cytokinin-induced bud initiation.

We also observed clear differences in response between cultivars. Of the six cultivars, only SC and SZ showed a significant increase in flower bud number after BA treatment (**Figure 2**). The flower bud number of SC increased from 3.14 in the control group to 5.29 in the BA-30I treatment group, whereas SZ showed an increase from 3.75 in the control group to 5.83 when treated with BA-30I. Moreover, the proportion of plants achieving high-quality grades for flower bud number increased.

The physiological basis of this variation was not investigated in this study. Several non-exclusive hypotheses are nevertheless available from the literature: differences in endogenous cytokinin homeostasis, in the abundance or affinity of cytokinin receptors, and in the efficiency of downstream signal transduction have each been proposed to modulate responsiveness to exogenous cytokinin in bulbous species (Shu et al., 2024). One hypothesis consistent with our phenotypic observations is that SC and SZ possess a cytokinin signaling capacity sufficient for exogenous BA to reach the threshold required for initiation of additional floral primordia, whereas the unresponsive cultivars do not. This hypothesis is not tested by the present data and is offered only as a direction for further work. Distinguishing among these possibilities would require quantification of endogenous cytokinin, gibberellin and abscisic acid concentrations in the shoot apex during the bud differentiation window, together with expression analysis of cytokinin receptor and response regulator genes.

Importantly, responsiveness to BA did not follow lineage. One of the two LA hybrids (SZ) and one of the two Oriental cultivars (SC) responded, while their respective counterparts (DU and AB) did not, so that responsiveness segregates within lineages rather than between them. Neither *L. longiflorum* cultivar responded, but with only two cultivars in that group this does not support a lineage-level inference, particularly as WT produced approximately two or fewer buds under all conditions and may simply lack the developmental capacity for additional primordia irrespective of cytokinin status. Shu et al. (2024) describe the cytokinin signaling machinery associated with meristem-derived organ initiation, including the biosynthetic and activating enzymes *LOG* and *CYP735A1*, the degradative enzyme *CKX*, the receptor histidine kinase *AHK2*, and the type-B response regulator *LlARR1*, which is expressed in the leaf axils of *Lilium lancifolium* during the preparatory phase of bulbil initiation. Since the abundance and activity of these components are under genetic control, variation among cultivars in any of them could in principle determine whether an exogenous cytokinin dose is sufficient to alter meristem fate. Our data are consistent with such variation operating at the level of the individual cultivar rather than the lineage, which has the practical implication that responsiveness must be established cultivar by cultivar and cannot be inferred from lineage.

One specific hypothesis worth testing is that the unresponsive cultivars maintain a higher endogenous abscisic acid concentration during the differentiation window. Abscisic acid antagonizes the initiation of meristem-derived organs in bulbous species, and the rate-limiting biosynthetic gene *NCED* together with the signaling components *PYL8* and *SAPK* is downregulated during initiation (Shu et al., 2024). If the same antagonism operates on floral primordium initiation, an elevated abscisic acid background could raise the cytokinin threshold required for a response and so account for the absence of an effect in AB, DI, DU and WT. This hypothesis is testable by quantification of abscisic acid in the shoot apex of responsive and unresponsive cultivars during the differentiation window, and we have not tested it in this study.

In contrast to SC and SZ, AB and DI had fewer than four flower buds on average under all treatments, and DU exceeded four only under the highest BA concentration, remaining below the premium criterion of five. These three cultivars therefore corresponded to medium-quality cut flowers irrespective of PGR treatment. WT had the lowest flower bud number among all cultivars (≤2), suggesting that this cultivar is not suitable for export. Within the range of treatments applied in this study, cultivar identity was therefore a stronger determinant of flower bud number than PGR treatment; the physiological basis of the insensitivity of WT was not determined.

### 4.3 Trade-off between current-season cut flower yield and stock bulb regeneration

The response of post-harvest bulb circumference to PGR contrasted with the response of flower bud number. Post-harvest bulb circumference was not significantly affected by PGR treatment in the analysis of variance (**Figures 1G, H**). In the principal component analysis, however, the control group was positioned furthest along the bulb circumference vector, and the separation increased with immersion time and concentration (**Figures 5C, D**). This suggests that a reduction may have occurred during BA treatment.

A plausible but untested explanation is competition for a shared assimilate pool. Sink establishment in bulbous species is closely tied to sucrose metabolism. During initiation of the bulbil primordium in *Lilium lancifolium*, sucrose synthase activity shifts towards the cleavage direction, lowering the local sucrose concentration, raising sink strength and drawing sucrose towards the developing organ, with starch synthesis following in the subsequent expansion phase (Shu et al., 2024). An equivalent mechanism has been described for floral bud differentiation in lily, in which the sucrose synthase genes *LoSuSy* and *LoSuSy4* promote differentiation through sucrose degradation (Pan et al., 2025). If BA treatment establishes additional reproductive sinks in the shoot by this route, a larger share of bulb reserves and current photosynthate would be diverted to the shoot, and less would remain for replenishment of the daughter bulb. Gibberellin, by contrast, is associated with expansion rather than initiation and with increases in bulb dimensions (Du et al., 2024; Shu et al., 2024), which is consistent with the trend towards larger post-harvest bulb circumference under GA+BA. We emphasize that carbohydrate partitioning was not measured in this study; direct verification would require quantification of starch and soluble sugars in bulb scales and shoot tissue across the season, together with sucrose synthase activity assays in the shoot apex during the differentiation window.

These results support a treatment strategy selected according to production objective. Where the objective is the current season’s cut flowers, immersion in 0.20 mM BA for 30 min is indicated, but only in cultivars demonstrated to be responsive. Where the objective is regeneration of stock bulbs for the following season, BA should be applied with caution, because a possible reduction in post-harvest bulb circumference cannot be excluded and was not resolved by the present single-season design. Bulb circumference is an indicator for evaluating stock bulb quality, as bulbs with larger circumferences produce more flower buds in subsequent growing seasons. Therefore, maintaining or increasing bulb size after harvest is crucial for sustainable lily production. The literature on bulbous species indicates that gibberellin promotes the expansion of storage organs (Du et al., 2024; Shu et al., 2024), and GA+BA was associated with a trend towards larger post-harvest bulb circumference in our study; however, this trend was not statistically significant, and we therefore present GA+BA for stock bulb production as a hypothesis to be tested rather than as a recommendation supported by the present data.

In the analysis by cultivar, BA treatment showed positive effects on SC and SZ. These cultivars exhibited the highest proportion of premium grades, implying their high suitability for export. However, among the quality-related traits, bulb circumference after harvest showed the lowest proportion of premium grades in SC. The SC cultivar showed an increased number of flower buds by utilizing nutrients in the bulb, but this could be a severe disadvantage in terms of cut flower production in the subsequent year. Therefore, when all quality-related traits are considered, SZ could be the most cost-effective choice for growers. SZ was the only cultivar that showed an increased number of flower buds when treated with PGR and maintained a large bulb circumference. The SZ cultivar is expected to be widely used in future programs to breed lilies suitable for export. This shows that, in responsive cultivars, BA treatment of small- and medium-sized bulbs can result in quality lilies comparable to those produced from large bulbs. Therefore, using small- and medium-sized bulbs, which are less expensive than the large bulbs, can provide sufficient economic value and represent a practical approach to reduce the burden of management costs on lily-producing farms.

### 4.4 Trait architecture, predictive modeling and the practical decision framework

We also investigated various traits and identified those strongly correlated with quality-related traits, and based on these findings, we developed machine learning models to predict quality-related traits and applied RSM to determine optimal conditions. PCA and correlation analysis identified shoot fresh weight and stem leaf number as the traits most closely associated with flower bud number among those that a grower can modify (Section 3.6). These traits are therefore interpreted as indicators of the growth state associated with a high bud number, rather than as determinants of it.

That these two traits emerged as the core predictors is consistent with the carbohydrate–hormone framework described for meristem-derived organ initiation in bulbous species. Initiation of such organs depends jointly on a hormonal signal that specifies meristem fate and on an assimilate supply sufficient to sustain the resulting sink; sucrose functions in this system both as substrate and as a signal, with soluble sugar associated with the initiation phase and starch with subsequent expansion (Shu et al., 2024). Stem leaves are the principal photosynthetic organs of the lily shoot and therefore determine the rate of carbon supply, while shoot fresh weight integrates the assimilate that has actually been accumulated. Together they index the carbon source available during the differentiation window, which the framework identifies as co-limiting with the hormonal signal. On this interpretation, exogenous BA can raise flower bud number only where assimilate supply is adequate, which would account for the cultivar-specific structure of the response surfaces in **Figure 8** and for the observation that BA increased bud number without increasing plant height or flower bud length. We note that this reasoning is inferential: neither photosynthetic rate nor carbohydrate content was measured here, and our data establish association rather than causation.

The contrast in accuracy between the two principal models should not be over-interpreted. The bulb circumference model includes post-harvest bulb weight among its predictors, a variable measured on the same organ at the same time and correlated with circumference at *r* = 0.933, so its accuracy largely reflects an allometric relationship rather than predictive capability. Beyond this, the two traits differ in their determination. Bulb circumference is a continuous size trait that integrates assimilate accumulation over the whole season and is therefore well predicted by other size variables. Flower bud number is a count trait fixed during a narrow differentiation window early in development, when it depends on the local hormonal state of the apex and on assimilate availability at that moment. Neither of these was measured, and the traits we did record integrate over the season rather than resolving that window. The moderate accuracy of the flower bud number model is therefore expected, and identifies the physiological measurements that would be required to improve it.

RSM was used to identify the optimal conditions for maximizing flower bud number for each cultivar. The results reflect cultivar-specific physiological characteristics and differences in the allocation of photosynthetically derived resources. Shoot fresh weight and stem leaf number are conventionally interpreted as integrative proxies for photosynthetic capacity and assimilate availability. Our data establish an association between these traits and flower bud number; they do not establish a causal role.

The divergent contour patterns in **Figure 8** indicate that the combination of assimilate supply and photosynthetic capacity associated with maximal bud number differs among cultivars. In AB, DU and SC the expected bud number was highest at the upper end of the stem leaf range, whereas in DI, SZ and WT it was highest at the lower end; and whereas SC required an intermediate shoot fresh weight to reach its maximum, SZ reached its own at the lowest fresh weight recorded. Within the carbohydrate–hormone framework, such differences would be expected if cultivars differ in the efficiency with which assimilate is converted into sink establishment at the apex, a step governed in bulbous species by sucrose synthase activity and by the cytokinin signaling components discussed above. A cultivar with efficient sink establishment would reach its bud number ceiling at a lower assimilate supply, whereas one with less efficient establishment would require a larger supply for the same outcome. This provides a coherent framework for the pattern, though it is not tested by our data, and confirmation would require parallel measurement of sucrose synthase activity and cytokinin signaling gene expression in the apex across cultivars. Differences in environmental responsiveness among cultivars may also contribute (Kim and Heo, 2025).

To make the operational value of the modeling explicit, we propose a two-stage decision framework. At the pre-planting stage, cultivar identity, bulb circumference and bulb weight are known, and the pre-planting model reported in Section 3.5 can be used to estimate whether a given lot is likely to reach the premium threshold and therefore whether BA immersion is economically justified for that lot. During the growing season, once stem leaf number is fixed and shoot biomass can be estimated, the cultivar-specific targets in **Table 3** and the surfaces in **Figure 8** allow the crop to be evaluated against its own cultivar’s optimum, so that lots can be allocated to export or domestic channels before harvest and fertigation and density decisions adjusted in subsequent cycles. The value of the framework lies in converting a large set of measured traits into a small number of cultivar-specific, agronomically interpretable targets, rather than in high-precision prediction of individual bud counts.

### 4.5 Limitations and directions for further work

Despite our findings, several limitations warrant consideration. As this study was conducted in a single growing season and post-harvest bulb quality was evaluated at a single time point, the long-term and cumulative impacts of repeated BA treatment across multiple seasons on bulb quality and subsequent flower production remain unclear. Although BA treatment increased flower bud number in the current year, a possible reduction in post-harvest bulb circumference could not be excluded.

This limitation is of particular concern for a treatment applied to the bulb itself. In bulbous species the storage organ integrates the assimilate balance of the preceding season, so a treatment that shifts allocation towards the reproductive shoot in one season alters the starting condition of the next. Our observation that BA increased flower bud number while reducing post-harvest bulb circumference would be the signature of such a shift, if confirmed. Whether the effect is self-limiting, so that a smaller bulb simply produces a smaller plant in the following season, or cumulative, so that repeated treatment progressively degrades the stock, cannot be determined from a single season and is the central open question raised by this study.

A two-year continuous cultivation trial is therefore in preparation. Bulbs of the responsive cultivars SC and SZ and of the unresponsive cultivar DU will be assigned in year one to a control, 0.20 mM BA, or GA+BA immersion, and the bulbs harvested from each treatment will be graded and replanted in year two under a factorial design in which the year-two treatment is crossed with the year-one treatment. This permits the cumulative effect of repeated BA application to be separated from the carry-over effect of a single application, and allows the practically important question of whether a BA-treated bulb can be rehabilitated by withholding treatment in the following season to be answered directly. Inclusion of an unresponsive cultivar allows any reduction in bulb circumference to be attributed either to the cost of additional bud production or to a direct effect of BA independent of bud number. Flower bud number, plant height, flower bud length and post-harvest bulb circumference will be recorded in both years, together with bulb starch and soluble sugar concentrations at harvest, so that the assimilate-partitioning interpretation advanced in Section 4.3 can be tested rather than inferred.

A structural limitation of the response surface analysis should also be acknowledged. Shoot fresh weight and stem leaf number are expressed during the growing season and are not available at planting, when the decision to apply BA must be taken. The response surfaces should accordingly be interpreted as cultivar-specific growth targets for in-season management rather than as pre-planting decision rules, and the pre-planting model reported in Section 3.5 was developed to address this gap. The accuracy of that model is nevertheless bounded, because bulb attributes explain only part of the variation in flower bud number. A genuinely predictive pre-planting decision tool would require physiological indicators of bulb status that were not measured here, in particular bulb carbohydrate reserves and the developmental stage of the scale meristem.

The moderate performance of the machine learning model for predicting flower bud number (R² = 0.558) reflects the complex interplay of genetic, environmental, and management factors. This study did not measure key physiological factors known to influence flower bud differentiation, such as carbohydrate content, endogenous hormone levels, and photosynthetic efficiency. Incorporating these physiological measurements along with environmental monitoring data in future studies would likely improve predictive accuracy and provide broader insights into the mechanisms underlying cultivar-specific responses to PGR treatment. The leave-one-cultivar-out results reported in Section 3.5 further indicate that the trait–bud number relationship does not transfer reliably to cultivars absent from the training data, so that any extension of this framework to additional cultivars will require those cultivars to be phenotyped rather than predicted.

## 5 Conclusion

This study examined whether pre-planting plant growth regulator treatment allows small- and medium-sized lily bulbs (14–16 cm) to produce export-grade cut flowers, and thereby reduces the economic burden associated with large bulbs. The central outcome is that treatment efficacy was strongly cultivar dependent. A 30-min immersion in 0.20 mM BA significantly increased flower bud number in two of the six cultivars examined, SC and SZ, in which the proportion of plants meeting the Japanese premium standard of at least five buds increased substantially. In the remaining four cultivars no significant increase was detected at any concentration or immersion time, and WT failed to meet export standards for either flower bud number or plant height under any treatment. GA+BA did not increase flower bud number in any cultivar. Responsiveness did not follow lineage, segregating instead within both the Oriental and the LA groups. These results should therefore not be read as a general recommendation for BA treatment in lily production. They indicate instead that pre-planting BA immersion is a viable cost-reduction strategy for cultivars that are demonstrably cytokinin-responsive, and that responsiveness must be verified for each cultivar, at pilot scale, before commercial adoption. No export quality trait other than flower bud number was significantly altered by PGR treatment, and a possible reduction in post-harvest bulb circumference under BA could not be excluded, raising the question of a trade-off between current-season cut flower yield and stock bulb regeneration; SZ was the only cultivar that combined an increase in flower bud number with maintenance of a large post-harvest bulb circumference and is accordingly the most cost-effective choice among those tested. Principal component and correlation analyses identified shoot fresh weight and stem leaf number as the growth traits most closely associated with flower bud number, and response surface methodology was used to convert them into cultivar-specific growth targets. The machine learning models are presented as trait-screening and grade-triage tools rather than as precision forecasting tools. Taken together, the study provides a cultivar-resolved framework that growers can apply to decide, for a given cultivar and bulb lot, whether small- and medium-sized bulbs can substitute for large bulbs.

## Data Availability

The raw data supporting the conclusions of this article will be made available by the authors, without undue reservation.
