# Supplementary material for: Effects of plant growth regulator treatments on quality-related traits and the establishment of cultivar-specific cultivation conditions in small- and medium-sized lily bulbs
Source: Front Plant Sci. 2026 Aug 19;17:1916678. doi: 10.3389/fpls.2026.1916678 (PMC13533972; doi:10.3389/fpls.2026.1916678)
Supplement: Supplementary file 1 [file SupplementaryFile1.docx]

***Supplementary Material***

Supplementary Figures

**Supplementary Figure 1**. Cultivar-specific responses of flower bud number to BA concentrations. **(A)** Aubisque (AB); **(B)** Difference (DI); **(C)** Ducati (DU); **(D)** Spring City (SC); **(E)** Sweet Zanica (SZ); **(F)** Woori Tower (WT). Different letters above the bars indicate significant differences among treatments within each cultivar (*p* < 0.05). Concentration treatments are labeled by PGR type and concentration level, followed by the letter C. BA, benzylaminopurine; PGR, plant growth regulators

**Supplementary Figure 2**. Cultivar-specific responses of flower bud number to GA+BA concentrations. **(A)** Aubisque (AB); **(B)** Difference (DI); **(C)** Ducati (DU); **(D)** Spring City (SC); **(E)** Sweet Zanica (SZ); **(F)** Woori Tower (WT). Different letters above the bars indicate significant differences among treatments within each cultivar (*p* < 0.05). Concentration treatments are labeled by PGR type and concentration level, followed by the letter C. GA, gibberellin; BA, benzylaminopurine; PGR, plant growth regulators

**Supplementary Figure 3**. Classification of quality grades according to the lily export quality standards for Japan. Flower bud number: premium (≥5 buds), good (4 buds), and medium (<4 buds); flower bud length: premium (≥12 cm), good (9.5–11.9 cm), and medium (<9.5 cm); plant height: premium (≥90 cm), good (80.1–89.9 cm), and medium (≤80 cm); bulb circumference: premium (>18 cm), good (14–18 cm), and medium (<14 cm). Concentration treatments are labeled by PGR type (BA or GA+BA) and concentration level (BA: 0, 0.10, 0.15, 0.20, 0.25 mM; GA+BA: 0, 0.07, 0.13, 0.20, 0.27 mM), followed by the letter C. The y-axis represents the frequency distribution of quality grades. GA, gibberellin; BA, benzylaminopurine; PGR, plant growth regulators

**Supplementary Figure 4**. Classification of quality grades according to the lily export standards for Japan. Flower bud number: premium (≥5 buds), good (4 buds), and medium (<4 buds); flower bud length: premium (≥12 cm), good (9.5–11.9 cm), and medium (<9.5 cm); plant height: premium (≥90 cm), good (80.1–89.9 cm), and medium (≤80 cm); bulb circumference: premium (>18 cm), good (14.0–18.0 cm), and medium (<14 cm). The x-axis represents different lily cultivars (AB, DI, DU, SC, SZ, and WT). The y-axis represents the frequency distribution of quality grades. AB, Aubisque; DI, Difference; DU, Ducati; SC, Spring City; SZ, Sweet Zanica; WT, Woori Tower

**Supplementary Figure 5**. Correlation matrix plot of correlation coefficients and corresponding significance levels between lily bulb characteristics. Bulb weight (BW), Bulb diameter (BD), Bulb height (BH), Bulb circumference (BC), Number of bulb roots (NBR), Root length (RL), Bulb weight after harvest (HBW), Bulb circumference after harvest (HBC), weight change of bulb (WC), and circumference change of bulb (DC). The diagonal boxes show the variable distributions split by cultivars. The lower boxes are composed of scatter plots, and the upper boxes show the Pearson correlation coefficients with significance levels (indicated by asterisks) for each cultivar. Each significance level is indicated as follows: p-value 0.001(***), 0.01(**), 0.05(*), and not significant (no asterisk). AB, Aubisque; DI, Difference; DU, Ducati; SC, Spring City; SZ, Sweet Zanica; WT, Woori Tower

**Supplementary Figure 6**. Correlation matrix plot of correlation coefficients and corresponding significance levels between phenotypic characteristics of lily bulbs. Variables include flower bud number (BUD), plant height (PH), fresh weight (FW), dry weight (DW), stem diameter (SD), number of stem leaves (NSL), leaf length (LL), leaf width (LW), flower length (FL), flower bud length (BL), petal length (PTL), petal width (PTW), sepal length (SL), sepal width (SW), filament length (FIL), days to flowering (DTF), and flower diameter (FD). The diagonal boxes show the variable distributions split by cultivars. The lower boxes are composed of scatter plots, and the upper boxes show the Pearson correlation coefficients with significance levels (indicated by asterisks) for each cultivar. Each significance level is indicated as follows: p-value 0.001(***), 0.01(**), 0.05(*), and not significant (no asterisk). AB, Aubisque; DI, Difference; DU, Ducati; SC, Spring City; SZ, Sweet Zanica; WT, Woori Tower

**Supplementary Figure 7**. Scatter plots of observed vs. predicted flower bud length (cm) based on datasets for **(A)** cultivar, **(B)** immersion time, and **(C)** concentration of PGR types. Each point represents an individual sample. The 1:1 slope is represented by a gray dotted line. PGR, plant growth regulators; RMSE, root mean square error; MAE, mean absolute error; GA, gibberellin; BA, benzylaminopurine; AB, Aubisque; DI, Difference; DU, Ducati; SC, Spring City; SZ, Sweet Zanica; WT, Woori Tower

**Supplementary Figure 8**. Scatter plots of observed vs. predicted plant height (cm) based on datasets for **(A)** cultivar, **(B)** immersion time, and **(C)** concentration of PGR types. Each point represents an individual sample. The 1:1 slope is represented by a gray dotted line. PGR, plant growth regulators; RMSE, root mean square error; MAE, mean absolute error; GA, gibberellin; BA, benzylaminopurine; AB, Aubisque; DI, Difference; DU, Ducati; SC, Spring City; SZ, Sweet Zanica; WT, Woori Tower

**Supplementary Figure 9.** Scaled variable importance for the machine learning models predicting **(A)** flower bud number and **(B)** bulb circumference after harvest. Importance values are scaled so that the most influential variable equals 1. For the bulb circumference model, post-harvest bulb weight (HBW) is the dominant predictor because it is measured on the same organ as bulb circumference; the model therefore chiefly recovers an allometric relationship. Post-harvest variables (HBW, HBC, WC, DC) were excluded from the flower bud number model as they are measured after the trait is determined. BC, bulb circumference before planting; BW, bulb weight before planting; BH, bulb height; BD, bulb diameter; NBR, number of bulb roots; RL, root length; PH, plant height; FW, shoot fresh weight; DW, dry weight; SD, stem diameter; NSL, number of stem leaves; LL, leaf length; LW, leaf width; HBW, bulb weight after harvest; Conc, concentration.

**Supplementary Figure 10.** Receiver operating characteristic (ROC) curves for discrimination of the premium grade (≥5 flower buds) by the machine learning models predicting flower bud number. The solid black line denotes the model trained on the full trait set (all vegetative and floral traits together with the treatment factors), and the dashed grey line denotes the model restricted to pre-planting information (cultivar, PGR type, concentration, immersion time, and the bulb attributes measured before planting). Sensitivity is plotted against 1 − specificity across all classification thresholds, and the diagonal line represents random discrimination (AUC = 0.5). The area under the curve (AUC) for the full trait set was 0.881 (95% CI 0.857–0.906); the pre-planting model gave a closely similar curve, indicating comparable discriminatory ability. AUC, area under the receiver operating characteristic curve; PGR, plant growth regulator.
